# Digital Learning Games for Mathematics and Computer Science Education: The Need for Preregistered RCTs, Standardized Methodology, and Advanced Technology

**DOI:** 10.3389/fpsyg.2020.02127

**Published:** 2020-10-15

**Authors:** Lara Bertram

**Affiliations:** ^1^MPRG iSearch, Max-Planck-Institute for Human Development, Berlin, Germany; ^2^School of Psychology, Faculty of Health and Medical Sciences, Guildford, United Kingdom

**Keywords:** game-based learning, active learning and teaching methodologies, academic emotions, academic motivation, STEM education, computational literacy, research practice

## Abstract

In today’s digital information society, mathematical and computational skills are becoming increasingly important. With the demand for mathematical and computational literacy rising, the question of how these skills can be effectively taught in schools is among the top priorities in education. Game-based learning promises to diversify education, increase students’ interest and motivation, and offer positive and effective learning experiences. Especially digital game-based learning (DGBL) is considered an effective educational tool for improving education in classrooms of the future. Yet, learning is a complex psychological phenomenon and the effectiveness of digital games for learning cannot be taken for granted. This is partly due to a diversity of methodological approaches in the literature and partly due to theoretical and practical considerations. We present core elements of psychological theories of learning and derive arguments for and against DGBL and non-DGBL. We discuss previous literature on DGBL in mathematics education from a methodological point of view and infer the need for randomized controlled trials for effectiveness evaluations. To increase comparability of empirical results, we propose methodological standards for future educational research. The value of multidisciplinary research projects to advance the field of DGBL is discussed and a synergy of Affective Computing and Optimal Experimental Design (OED) techniques is proposed for the implementation of adaptive technologies in digital learning games. Finally, we make suggestions for game content, which would be suitable for preparing students for university-level mathematics and computer science education, and discuss the potential limitations of DGBL in the classroom.

## Introduction

Mathematical and computational skills have become an integral component of basic literacy, and improving students’ proficiency in mathematical and computational thinking plays a key role in many countries’ education strategies (Committee on STEM Education, 2018; European Schoolnet, 2018).

Yet, while over the last decades Organisation for Economic Co-operation and Development (OECD) countries’ expenditure per student increased on average by 15%, students’ performance did not change significantly (OECD, 2019b). Pertaining issues are the significant and robust relationship between socioeconomic status and academic performance, especially in science and mathematics (Thomson, 2018; OECD, 2019b), a negative association between countries’ socioeconomic inequality and performance in the Programme for International Student Assessment (PISA; Parker et al., 2018), and decreasing mobility between socioeconomic backgrounds (OECD, 2018). Students generally tend to lose motivation, competency beliefs, and interest along the educational chain (Wigfield et al., 1991; Jacobs et al., 2002; Frenzel et al., 2010), which in turn affects academic performance (Singh et al., 2002; Arens et al., 2016) and course selection (Köller et al., 2001). Accordingly, educational interventions are needed which effectively decrease achievement gaps, sustain motivation, engagement, and interest in mathematics and computational subjects and provide educational opportunities which all students profit from (van den Hurk et al., 2019).

Playful learning (Hirsh-Pasek et al., 2009) has long been advocated as a promising pedagogical approach for effectively teaching students mathematics and computer science in an engaging, fun and motivating way (Mayo, 2009; Papastergiou, 2009; Zosh et al., 2016). Game-based learning interventions are supposed to offer students active self-guided learning opportunities and positively affect attitudes, emotions, motivation, and engagement (Vandercruysse et al., 2012; Weisberg et al., 2016).

With PISA’s focus for 2024 being on the “ability of students to learn in a digital world,”^1^ the importance of digital learning, including digital game-based learning (DGBL), can be expected to rise. The anticipated benefits of digital over non-digital learning tools lie in their flexibility, adaptiveness, and interactivity which foster non-linear and self-directed (no preset order, students can actively choose the next step) learning (Hsiao et al., 2010; Brusilovsky, 2012; Kärkkäinen and Vincent-Lancrin, 2013; Committee on STEM Education, 2018). Yet, to ensure the effectiveness of digital learning, the design and development of digital learning environments should be evidence-based and grounded on psychological theory. Furthermore, rigorous scientific evaluations of digital learning tools are required to systematically assess their relative effectiveness regarding learning outcomes and psychological effects (Kickmeier-Rust et al., 2006; Kickmeier-Rust and Albert, 2010; Nussbaumer et al., 2019). In the following, we briefly review key psychological literature on the relationship between emotion, motivation, mode of information acquisition, and learning. Based on the reviewed evidence, we develop our arguments for and against DGBL, infer the need for interdisciplinary research and advanced technology, and propose methodological standards for effectiveness evaluations.

## Psychological Theory of Learning

Learning-related cognitive, motivational and emotional processes shape the learning process (Arens et al., 2016; Pekrun et al., 2017), as well as the way information is acquired (Bruner, 1961; Schunk, 1990; Gureckis and Markant, 2012; Ruggeri et al., 2019). These variables are closely interrelated and significantly shape the learning process. Thus, they deserve special consideration in any educational setting.

### Academic Emotion

Academic emotions are defined as emotions students experience in an academic setting, i.e., emotions associated with achievement, instruction, and the learning process (Pekrun et al., 2002, 2017). Mathematics emotions are closely related to mathematics achievement: over a 5 years period of annual testing (grades 5–9), mathematics performance, measured by end-of-the-year grades and standardized test scores, and mathematics emotions, measured with the Achievement Emotions Questionnaire (AEQ)-Mathematics (Pekrun et al., 2011), reciprocally affected each other (Pekrun et al., 2017). Mathematics anxiety has been consistently shown to be negatively associated with mathematics performance, with effect sizes being moderate (Ma, 1999; Namkung et al., 2019).

Emotions have a subjective, a cognitive and a behavioral component. Due to their complexity, they often cannot be pinpointed to one concrete sensation. For example, when working on a challenging task, students can be anxious that they might fail, motivated to master the challenge, and proud when they master sub-goals – all at the same time. Given that emotions are associated with other learning-relevant psychological resources such as motivation, attitudes, and interest, stimulating positive academic emotions, accurately detecting students’ emotions, and reacting to them appropriately are of crucial importance in educational settings.

### Emotion and Motivation: Control Value Theory of Achievement Emotions

Control value theory of achievement emotions describes the relationship between academic emotions and motivation (Pekrun et al., 2007a; Pekrun and Stephens, 2010). Students’ expectations, attributions, and competency beliefs influence their perceived control, which evokes an emotional reaction. For example, when being asked a question by the teacher, low perceived controllability of the situation may arise from the belief that one is not talented in mathematics. This creates the expectation of being unable to answer the question correctly, which in turn may evoke anxiety, helplessness, or sadness. In contrast, when feeling in control, a student may enjoy the opportunity to answer a question and be the focus of attention. The perceived value of an academic activity shapes the strength of the experienced emotion. For example, a high or low test score in a mathematics exam may not evoke strong emotions in a student who thinks that mathematics is not important for her future life, in contrast to a student who values mathematics very highly.

### Active Learning and Flow Theory

Active learning environments give students the opportunity to self-regulate, develop intrinsic motivation, and exert control over the learning process (Bandura, 1991; Zimmerman et al., 1992; Zimmerman, 2002), which are beneficial for children’s psychological development and learning outcomes (Bruner, 1961; Kolb, 1984; Boekaerts, 1997). From a cognitive and computational perspective, active information acquisition and control over the flow of incoming information positively affect efficiency of information acquisition, learning, and memory (Gureckis and Markant, 2012; Ruggeri et al., 2019). Flow theory (Csikszentmihalyi, 1975; Csiksentmihalyi and Schiefele, 1993) states that intrinsically motivated behavior and the experience of *flow* are fostered in situations, which are shaped by a learner and characterized by a fit between learners’ abilities and the demands of a situation.

### A Psychological Argument for Game-Based Learning

This brief discourse into the psychology of learning elucidates the complex interrelation between characteristics of the learning environment, students’ academic motivation and emotions, and learning outcomes. Well-designed learning games are interactive learning environments which give students the opportunity to acquire knowledge and practical skills in a playful and self-directed way, experience engagement and flow and develop positive attitudes, feelings, and competency-beliefs (Gee, 2008; Kapp, 2012; Plass et al., 2015; Weisberg et al., 2016; Becker, 2017). Digital learning games are expected to even expand these positive characteristics of learning games, given their high flexibility, engagement, and fun due to their digital nature (Prensky, 2003). Yet, to successfully exploit the psychological, pedagogical, and academic potentials of games in DGBL environments, not only a firm grounding in psychological and pedagogical theories (Malone, 1981; Ryan et al., 2006; Starks, 2014) but also adherence to standards in digital educational game design (Göbel et al., 2018), advanced technologies and rigorous effectiveness evaluations are of fundamental importance. In the following, we discuss previous literature on DGBL and make methodological suggestions for future research in the field. We stress the need for interdisciplinary research projects and advances in technology research, especially for implementing adaptivity in learning games. We also highlight possible limitations of DGBL and suggest ways to overcome these limitations.

## Digital Game-Based Learning Research: Current Practice and Future Developments

Research on DGBL paints a complex picture: it is generally characterized by a multitude of approaches, terminologies, and methodologies (Connolly et al., 2012; Boyle et al., 2016; de Freitas, 2018). While some studies report overall positive effects of digital game-play on learning outcomes (Chang et al., 2015) and motivational variables (Hung et al., 2014; Partovi and Razavi, 2019), others report no general advantage of digital games over standard teaching methods (Ke, 2008a; Brom et al., 2011). In the context of mathematics education, Erickson (2015) evaluated 30 digital mathematics games and found that only five scored high on all the three identified motivational dimensions (ease of understanding, control, and immersion). The investigated games differed in the degree to which they provided cognitive scaffolding and offered opportunities for proficiency development and reflection upon learning strategies. In a recent meta-analysis which included 17 studies, Byun and Joung (2018) found an overall weighted effect size of *d* = 0.37 for the relative effectiveness of digital games for learning mathematics. Yet, effect sizes vary largely between the analyzed studies. For example, while an effect size as small as *d* = 0.13 was reported by Ke (2008b), very high effect sizes above two were found in two studies by Sedig (2007, 2008) and a set of experiments by Shin et al. (2012). Besides these extreme cases, the remaining studies found small (van Eck, 2006; Ke and Grabowski, 2007; Ke, 2008a; Bai et al., 2012; Kolovou et al., 2013; Lin et al., 2013; van den Heuvel-Panhuizen et al., 2013; Pareto, 2014; Bakker et al., 2016), medium (Kebritchi et al., 2010; Plass et al., 2013) or large (Sedig, 2007; Yang and Chen, 2010; Ke, 2013) effects.

The high variability of results in research on DBGL in mathematics education is indicative of differences in research methodologies and practices, which makes general conclusions about the effectiveness of DGBL in mathematics difficult. Among the most striking differences between studies are design and content of the games used, research designs (RCT or quasi-experiment; mixed or quantitative methods), age groups (primary, secondary, or university education), and number of participants as well as effectiveness criteria and instruments employed in the effectiveness evaluation. The most prevalent research design is the quasi-experiment; less often randomly controlled experimental designs are realized (Boyle et al., 2016). Group assignment is usually conducted on a class level (Papastergiou, 2009; Kebritchi et al., 2010; Bai et al., 2012; Kim et al., 2017; Brezovszky et al., 2019) and seldom on a school level (Rutherford et al., 2014), and very few studies follow an experimental approach with randomization on subject level (Plass et al., 2013). Whereas most studies include a control group, studies without a control group can also be found (for example, Iten and Petko, 2016). Often multiple measurement points are reported, differing in time intervals between measurements (Bottino et al., 2007; Kebritchi et al., 2010; Habgood and Ainsworth, 2011; Bai et al., 2012; Shin et al., 2012; Bakker et al., 2015). Methodologies entail qualitative, quantitative, and mixed methods, with the latter two being the most prevalent (for a comprehensive overview, see Byun and Joung, 2018).

### The Need for Preregistered Randomly Controlled Trials, Standardized Procedures, and Methods

Even though quasi-experimental research designs and randomization on a class level may be the most feasible approach for educational research, randomly controlled experiments with randomizing on a subject level are fundamental for generating solid empirical evidence. Preregistering experiments (or even using preregistered reports) increase credibility of results and limit questionable research practices (Nosek et al., 2018). Furthermore, standardizing pre‐ and post-test measures raises comparability between studies. We suggest using standardized scales from the international studies PISA and TIMMS if applicable (International Association for the Evaluation of Educational Achievement, 2015; OECD, 2019a) and standardized psychological instruments, for example, scales measuring academic emotion (Pekrun et al., 2011; Lichtenfeld et al., 2012), self-concept (Pekrun et al., 2007b; Arens et al., 2016), and motivation (Schwarzer and Jerusalem, 1995; Midgley et al., 1998). For evaluations of the relative effectiveness of digital learning games for learning outcomes, standardized tests are not always available. These tests should then be developed in collaboration with experts (e.g., cognitive scientists, psychologists, or teachers), validated, and tested for reliability. To further standardize timing of measurements, we suggest conducting the pre-test a week before the intervention to avoid effects of testing on experimental results and to generate a non-biased baseline. The post-test is conducted on the day of the intervention in case of a single intervention to measure immediate effects. In case of a longitudinal study, it may be advisable to have measurements on each intervention day as well as one day after the intervention is completed to balance out daily variability. Follow-up tests are important to evaluate the persistence of effects; their timing depends on the study design and the resulting shape of the forgetting curve, as well as the claims authors make regarding the effectiveness of their intervention (Murre and Dros, 2015; Nussbaumer et al., 2019). Enriching quantitative measures with qualitative measures and classroom discussion can be informative to determine the feasibility of a method, better understand the underlying mechanisms, and solidify students’ learning, yet the core criterion in effectiveness evaluations should be preregistered statistical analyses of experimentally obtained data.

### Interdisciplinary Research on Adaptive Game-Based Learning

A promising way to improve learning experiences in digital learning environments is adaptive technology. Adaptive learning tools promise to offer students the learning experiences they need in a given moment by recognizing their cognitive, motivational, and emotional states. International and interdisciplinary research on evidence-based digital education platforms which adapt to students’ individual needs is growing. Projects range from adaptive structuring of learning experiences on digital learning platforms (Hsiao et al., 2010; Brusilovsky, 2012) to adaptive DGBL interventions (Brezovszky et al., 2019), developing sophisticated software components for adaptive learning based on sound psychological and pedagogical principles (Kickmeier-Rust et al., 2006; Maurer et al., 2017; Nussbaumer et al., 2019). The authors distinguish different levels of adaptivity and corresponding software assets:

1. Pre-game adaptation: personalization of the initial stages of the game based on student characteristics, which are measured prior to game-play using standardized instruments.
2. Competence-based in-game adaptivity: monitoring learning progress to adapt learning path, instructions, and support.
3. Psychological in-game adaptivity: monitoring psychological state and adapting game characteristics accordingly (e.g., adapt difficulty level, offer support, and change game design).

As the body of research on adaptive digital learning games is growing, meta-analyses are needed to determine the relative effectiveness of different kinds of adaptivity, e.g., based on performance, motivation and/or emotion, adaptation of game design, instruction, and/or game content. Importantly, adaptive learning games, which are currently available online, are not necessarily scientifically evaluated, and teachers and parents should be made aware of this. A way to give users guidance would be a quality seal, which indicates the level of scientific evidence (research methodology, see “The Need for Preregistered Randomly Controlled Trials, Standardized Procedures, and Methods,” and outcomes) for the effectiveness of an adaptive digital learning game.

### Affective Computing and Optimal Experimental Design for Software Adaptivity

One research stream on adaptive digital learning is based on insights from Affective Computing. Affective Computing is defined as “computing that relates to, arises from, or influences emotions” (Picard, 1997, p. 1). It is a relatively young field of research, yet it has rapidly grown over the last decades (Picard, 2015). A recent systematic review (Aranha et al., 2019) revealed that education is the most frequent application area of Affective Computing. A majority of studies investigate affectively adaptive digital games, yet affective learning (Picard et al., 2004) also refers to affectively intelligent tutoring, dialogue, agent-based, and other learning systems (Santos, 2016).

The general goal of affective learning research is to develop software which recognizes users’ affective state and adapts its interactive behavior accordingly, based on sophisticated models of emotion-cognition interaction (D’Mello et al., 2008; Hudlicka, 2008, 2017; Cooper et al., 2011; D’Mello and Graesser, 2015). Despite the theoretical complexity and methodological difficulties in emotion research, advances have been made in the modeling of emotion-cognition interactions (Hudlicka, 2011, 2017) and the development of formal emotion languages (Schröder et al., 2015). The methodologies used for emotion detection include psychophysiological methods (electrodermal activity, heart rate recording, EEG, and EMG measures), camera-based methods (capturing facial expressions, eye-movements, and voice), and behavioral measures (user input and in-game behavior). Emotionally adaptive learning games promise to offer students learning experiences which are tailored to their emotional needs. Yet, emotional adaptivity must be handled with care: adaptivity requires the collection of sensitive data, which may or may not be adequate in a given context. Due to the still low accuracy in emotion detection, predictions may be inaccurate (Aranha et al., 2019), indicating the need for further advances in the development of non-intrusive and reliable emotion detection mechanisms. This also requires improved software infrastructure for interoperability between systems, adequate and contextual feedback, and interaction mechanisms (Santos, 2016). Lastly, educators may prioritize giving students the option to experience a wide range of situations and emotions, including those which are not adapted to their learning profile. Keeping these considerations in mind, how can adaptive technology be enhanced?

Computational methods which have previously been employed to implement adaptivity are supervised classification, probabilistic models, and regression analyses (Santos, 2016). We propose Optimal Experimental Design (OED), a computational method which optimizes experimental designs for discrimination among multiple psychological models (Myung and Pitt, 2009), as a novel tool for effectively implementing software adaptivity in learning games. Game-play situations can be regarded as mini-experiments, and their outcomes can inform the system’s knowledge base about the user. OED confronts the learner with those situations which are most informative for the system’s construction of the learner model. It can be integrated into the system’s profiling asset (Maurer et al., 2017) and support in-game adaptivity based on performance, motivation, engagement, and emotional state of the learner, allowing the system to build an increasingly fine-grained model of the learner and personalize learner-system interactions. A python package, ADOpy (Yang et al., 2019), is available as an open source resource to the public^2^.

### Computational and Mathematical Topics for Game-Based Primary Education

Even though the number of digital educational games for learning mathematics (Erickson, 2015; Byun and Joung, 2018) and programming (Lindberg et al., 2018) has been growing, evidence-based digital learning games for computer science in primary education are rare. In a recent systematic literature review, only two studies were identified which investigated DGBL in elementary computer science education, both of which were of relatively low quality in terms of study design, appropriateness of methods and analyses, generalizability, relevance, and trustworthiness of findings (Hainey et al., 2016).

The university guidelines for undergraduate computer science curricula from ACM and IEEE (2013) include the following topics, which we suggest for game-based learning in primary and secondary education and which have already been successfully implemented in games: basic principles of machine learning (Wallace et al., 2008; Stöckl, 2019), algorithms and complexity (Hong and Kung, 1981; Battistella et al., 2017), information theory (Greeff et al., 2017), and computer architecture (Tlili et al., 2016). In mathematics education, the majority of learning games focus on numbers and operations, algebra, geometry, measurement, and data analysis and probability (Byun and Joung, 2018). Additional topics for game-based learning in mathematics are combinatorics, probabilities, functions, and number systems. Besides educational content, the so-called “21st century skills” (Binkley et al., 2012), which include critical thinking skills such as scientific reasoning, systems thinking, computational thinking, decision making, and problem solving, can be taught in a gamified way (Qian and Clark, 2016).

**Figure 1:**
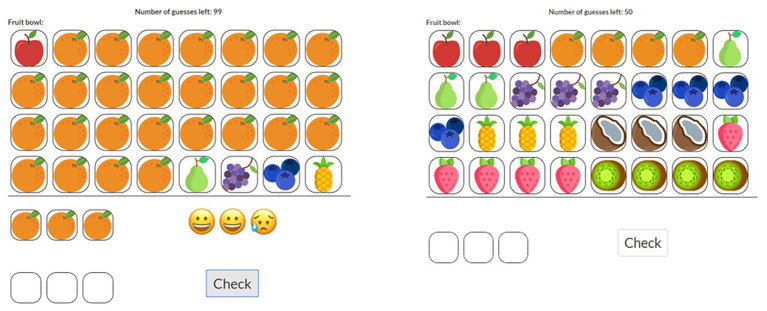
Icon arrays representing two example code jars (in this version of the game fruit bowls) which generated the secret code. **Left panel**: low entropy code jar. The first guess and the corresponding feedback are displayed. Happy emoticon: correct fruit and correct position; neutral emoticon: correct fruit but wrong position; sad emoticon: incorrect fruit and position. Positions of faces do not correspond to positions in the code. **Right panel**: high entropy code jar. Game environment before the first guess was entered. Initially, each position of the code is blank, and players can cycle through the fruits by clicking on the blank field. Feedback is provided after players clicked on the “Check” – button. Play the game yourself: http://jonathandnelson.com/curious/masterminding.html.

**Figure 2:**
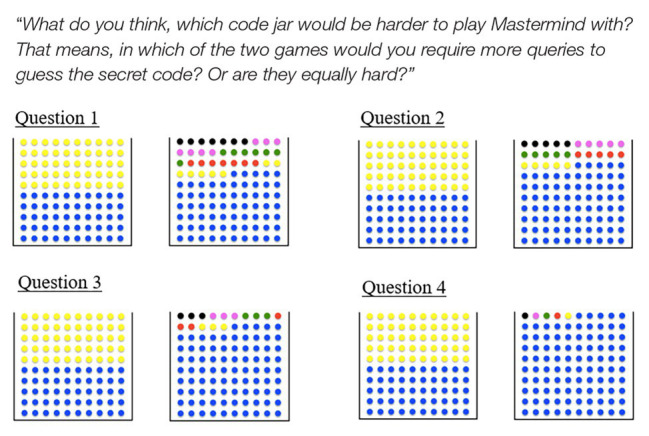
Example pre‐ and post-test questions testing entropy intuitions. Students are asked for each pair of code jars which of the two would be harder/easier to play with or whether the two urns are equally hard. Answers to these questions quantify entropy intuitions (Crupi et al., 2018).

We are currently developing a game, Entropy Mastermind (Figure 1; Schulz et al., 2019), to promote students’ entropy intuitions by providing experiential access to the relationship between probability distributions and the mathematical concept entropy (Crupi et al., 2018). Entropy is not only an important concept in cognitive science, computer science, mathematics, the philosophy of science, and information theory but it also has many practical applications (Martignon et al., 1991; Mana et al., 2018) and educational relevance (Haglund et al., 2010). The game Entropy Mastermind is an extension of the classic Mastermind game. In Entropy Mastermind, a secret code is generated from a probability distribution by random drawing with replacement. The player (code breaker) has to guess the secret code by testing out codes and getting feedback about the correctness of the guessed code. The feedback is comprised of three different kinds of smileys: a happy smiley indicating that a guessed item is correct in kind and position, a neutral smiley indicating that a guessed item is the correct kind but not in the correct position, and a sad smiley indicating that a guessed item is incorrect regarding both kind and position. Importantly, the order of smileys in the feedback is always the same: happy smileys come first, then neutral, and lastly sad smileys – the position of smileys in the feedback array does not correspond to the positions of items in the guessed code. The entropy of the distributions from which the secret code is generated varies between rounds of the game. Figure 2 displays a low entropy (left game environment) and a high entropy (right game environment) Entropy Mastermind game. The level of entropy in the underlying probability distribution affects the difficulty of the game (the number of queries needed to guess the secret code; Schulz et al., 2019), and the resulting variations in difficulty give experiential access to the concept entropy.

In the Entropy Mastermind educational intervention, learning about entropy is evaluated using specifically designed test items which quantify entropy intuitions (for example, Figure 2); psychological effects are assessed using the AEQ (Lichtenfeld et al., 2012), the mathematical self-concept scale (Pekrun et al., 2007b; Arens et al., 2016), and the general self-efficacy scale (Schwarzer and Jerusalem, 1995). First studies using Entropy Mastermind in educational contexts have been conducted: these include the development and implementation of a roadmap for an instructional unit aimed at fostering elementary students’ intuitions about entropy using a non-digital version of Entropy Mastermind (Özel et al., n.d., submitted). Based on insights from this first study, a digital version of Entropy Mastermind was developed (Figure 1), and first pilot studies conducted using this digital version of the game (Bertram et al., 2019; Schulz et al., 2019; Bertram et al., 2020). Yet, further research is needed to evaluate the effect of playing Entropy Mastermind on entropy intuitions, knowledge about probabilities, and learning-related psychological variables, and to further validate the developed test items for assessing entropy intuitions.

### Possible Limitations of Digital Game-Based Learning

Despite the above described potential benefits of DGBL, it is important to also consider its limitations. Digital worlds are reduced in their dimensionality compared to the physical world, lacking sensual experiences such as touch or smell. Embodied education (Shapiro and Stolz, 2019), an emerging research field rooted in the literature on grounded and embodied cognition (Varela et al., 1991; Clark, 1996; Barsalou, 2008; Barsalou, 2010; Shapiro, 2019), education theory, and learning science (Montessori, 1972; Bresler, 2005), stresses the fundamental role of bodily experiences in the learning process (Hostetter and Alibali, 2008; Tellier, 2008). In digital learning games, students remotely interact with the game environment by touching a display, using a keyboard, mouse, or voice control. This kind of interaction is indirect and mediated (the digital device is the mediator) compared to interactions in physical environments. Physical behavior may not only be reduced to finger, hand, or arm movements, but also be incongruent to the actual behavior carried out in the game environment. This divergence between cognition and behavior may interfere with the learning process (Shapiro and Stolz, 2019). Yet, digital learning games may overcome these limitations by incorporating embodiment principles in the game design (Black et al., 2012): gestural or natural user interfaces can be operated *via* touch (touch use interfaces) or remotely (free form interfaces), stimulating body movements congruent to the learning content, and thus benefitting learning (Hostetter and Alibali, 2008; Tellier, 2008). For example, Wang et al. (2014) successfully created a natural user interface, operated *via* body movements, to teach elementary students the projectile motion.

Other limitations arise from the potentially high costs associated with digital game design and the purchase of digital technologies. These costs are justifiable under the assumption that digital learning games significantly improve education. Digitalizing education is also a necessary step toward modernization and improvement of the education system. Yet, in the process of introducing digital learning tools into the classroom – including digital learning games – it is important to realistically assess the relative benefits of these digital learning games and conduct cost-effectiveness evaluations (Tobias et al., 2014). If, for example, an adaptive game turns out to only have little advantage (e.g., regarding learning outcomes or effects on academic emotion and motivation) over its non-adaptive version, the development costs may exceed the benefits. Similarly, a digital learning game may or may not be more effective for learning than its non-digital version. In these cases, it is advisable to consider the use of relatively cost-effective methods to enrich education with games, e.g., using haptic versions (to reduce costs associated with purchasing digital devices) or already programmed digital versions of classic games (to reduce game development costs), such as chess, card games, riddles, board games, code-breaking games, or puzzles. These games are engaging, intrinsically motivating, and fun to play but do not need sophisticated visuals and complex virtual environment simulations.

Also, it should be carefully observed if using digital games in education disadvantages those students who have limited financial capacities and may not have access to digital devices at home. Equal opportunities are a key characteristic of good education systems and must be constantly preserved and improved. Another delicate issue associated with digital learning is students’ digital rights: every student and/or their parents or legal guardians should own their data and be able to decide how their data are used, for example, by giving informed consent about the usage of their data or by having access to their own data *via* a password. When collecting data is part of digital game-based education interventions, ethical integrity, thoughtful data handling, and strict adherence to data protection regulations are a prerequisite and must be accompanied with transparent communication with parents or legal guardians.

## Discussion

In this article, we discussed future directions in research on DGBL in mathematics and computer science education. We highlighted the importance of a sound psychological foundation for the development of learning games and the need for interdisciplinary research projects and randomized controlled experimental designs to evaluate the effectiveness of games and game features. We introduced a new methodology to implement adaptivity, a synergy of Affective Computing and OED techniques and suggested topics for digital mathematics and computer science games. We also presented our own digital educational game, Entropy Mastermind, for fostering students’ intuitions about entropy. Lastly, we discussed limitations of DGBL and suggested ways to overcome potential complications. When keeping in mind these potential limitations and complications, game-based digital and non-digital learning is a fruitful field for systematic interdisciplinary research and a promising practical educational tool for enriching educational methods and realizing equal opportunities in classrooms of the future – giving all students the opportunity to learn at their best.

## Author Contributions

LB conducted the literature review and conceptual analysis, wrote the first draft of the manuscript, revised, read, and approved the submitted version.

### Conflict of Interest

The author declares that the research was conducted in the absence of any commercial or financial relationships that could be construed as a potential conflict of interest.

## Footnotes

^1^http://www.oecd.org/pisa/

^2^https://github.com/adopy/adopy
